# Comprehensive network modeling from single cell RNA sequencing of human and mouse reveals well conserved transcription regulation of hematopoiesis

**DOI:** 10.1186/s12864-020-07241-2

**Published:** 2020-12-29

**Authors:** Shouguo Gao, Zhijie Wu, Xingmin Feng, Sachiko Kajigaya, Xujing Wang, Neal S. Young

**Affiliations:** 1 Hematopoiesis and Bone Marrow Failure Laboratory, Hematology Branch, NHLBI, National Institutes of Health, Bethesda, MD 20892 USA; 2 Division of Diabetes, Endocrinology, and Metabolic Diseases (DEM), NIDDK, National Institutes of Health, Bethesda, MD 20817 USA

**Keywords:** Hematopoiesis, Gene regulatory network, Co-expression network, Single-cell RNA sequencing, Cross-species network analysis

## Abstract

**Background:**

Presently, there is no comprehensive analysis of the transcription regulation network in hematopoiesis. Comparison of networks arising from gene co-expression across species can facilitate an understanding of the conservation of functional gene modules in hematopoiesis.

**Results:**

We used single-cell RNA sequencing to profile bone marrow from human and mouse, and inferred transcription regulatory networks in each species in order to characterize transcriptional programs governing hematopoietic stem cell differentiation. We designed an algorithm for network reconstruction to conduct comparative transcriptomic analysis of hematopoietic gene co-expression and transcription regulation in human and mouse bone marrow cells. Co-expression network connectivity of hematopoiesis-related genes was found to be well conserved between mouse and human. The co-expression network showed “small-world” and “scale-free” architecture. The gene regulatory network formed a hierarchical structure, and hematopoiesis transcription factors localized to the hierarchy’s middle level.

**Conclusions:**

Transcriptional regulatory networks are well conserved between human and mouse. The hierarchical organization of transcription factors may provide insights into hematopoietic cell lineage commitment, and to signal processing, cell survival and disease initiation.

**Supplementary Information:**

The online version contains supplementary material available at 10.1186/s12864-020-07241-2.

## Background

Hematopoietic stem cells (HSCs) are characterized by their ability to extensively proliferate, differentiate into diverse mature blood cells, and self-renew to maintain the stem cell pool [1, 2]. Imbalanced and aberrant HSC differentiation lead to biased production of cell types and underlie many constitutional and acquired blood diseases. Differentiation versus maintenance, proliferation versus quiescence, lineage specificity and maturation of cells are mainly determined by transcription factors (TFs) and their target genes (TGs) within complex transcriptional regulatory networks. Experimental identification of transcriptional regulation is challenging, and many computational network reconstruction methods have been developed in order to infer functional relationships between gene pairs and to provide indirect evidence of transcriptional regulation.

Most network reconstruction strategies in the past have been based on measurements of transcription in bulk cell samples, in which the data represents an average of gene expression patterns across thousands to millions of cells [3, 4]. Recently, single-cell RNA-sequencing (scRNA-seq) has developed as a powerful discovery tool to characterize global regulatory programs in hematopoiesis [5]. The opportunity exists to derive a global regulatory network because the number of observations in a typical single-cell experiment is generally much higher, and thus provide far more and also different information than can be obtained from experiments in bulk population [6]. Jie Wang et al. found that bulk sample specific co-expressed genes were slightly enriched for protein interactions in BioGRID (https://thebiogrid.org) with 1.6-fold enrichment. In contrast, interactions were much more enriched in single-cell specific co-expressed genes with a 5-fold enrichment compared to the expectation [7]. Compared with bulk expression data, the co-expressed genes in single cells encode proteins that are more likely to physically interact with each other. However, single-cell data are limited due to dropout events (expressed genes undetected by scRNA-seq) and noise (technical issues such as PCR amplification bias [8]), which often make reliable inferences of regulatory networks difficult. Dropout events are most important in affecting performance when co-expression metrics are directly calculated using normalized expression data represented by read counts [9, 10]. Many algorithms have been developed to impute dropouts but the inferred correlation can be highly skewed to positive values and affected by the choice of parameters, as occurs with Markov Affinity-based Graph Imputation of Cells (MAGIC) [10]. The algorithm bigScale2 is advantageous as it clusters cells and calculates z-scores for each gene in terms of differential expression between pairs of clusters, and then uses z-scores to calculate gene-pair correlations [9]. bigScale2 circumvents dropout and can detect gene-to-gene correlations that are often otherwise missed.

In a co-expression network, not all genes are equal in their influence on the network stability and robustness (Figure S1A). A single gene’s importance in the network context can be determined using its centrality measures, such as “connectivity”, also called degree (number of directly connected genes), “betweenness” (frequency of being passed by the shortest paths of pairs of all other genes), “clustering coefficient” (probability of connections among a gene’s direct neighbors) and “PageRank” (popularity of a gene based solely on the number of its interactions [9]). Genes of high centrality tend to be essential genes, required for cell survival; several typical genes with high centralities are shown in Figure S1A. The yeast co-expression network has been found to possess a “small-world” and “scale-free” architecture. Small world networks contain highly connected subnetworks, and thus have higher clustering coefficients (G21 in Figure S1A). In a scale-free network, a majority of the genes have one or two connectivities, and only a few genes have a large number of connectivities.

Presently there is no published comprehensive analysis of regulatory networks during hematopoiesis. In regulatory networks, edges connecting gene pairs are directed, from TFs to target genes. In graph theory, such networks are called directed networks. Gene regulatory networks (GRNs) appear to share structural characteristics with social networks, such as governmental and corporate organizations that are more oriented toward control than to communication [11, 12]. Hierarchical structures do exist in biological regulatory networks such as yeast and *E. coli* [13].

A typical GRN usually has multi-level hierarchical layers (Figure S1B). For yeast, TFs at the top, middle and bottom levels are related to different biological themes. Top level TFs have more protein-protein interactions, the middle layer genes tend to be collaborative in that targeted genes are co-regulated by other TFs, and lower level genes tend to be essential. Network motifs, defined as over-represented subgraphs, are widely considered to be functional units of GRNs. Two common motifs are feed-forward loop (FFL) and bi-fan (both color-highlighted in Figure S1B). In FFL, a TF (B3) regulates another TF (C4) and they together co-regulate a third gene (D4). A bi-fan motif includes two TFs (A1, A2) that both regulate on the same two genes (B1, B2). Characterization of the GRN hierarchical structure and its motifs as the smallest structural and functional units should be useful in characterization of the transcription programs in normal cellular development and function, and in disease.

Animal models are widely used on the predicate that fundamental biochemical processes are conserved across species, most commonly between human and mouse, an assumption not always supported by rigorous or systematic analyses [14–16]. Evolutionary cross-species comparisons can provide a framework to refine human biological research [17]. scRNA-seq has been extensively applied to study hematopoiesis of human and mouse, but cross-species comparison of the hematopoietic system is not firmly established without published network-level comparisons [17].

Here we apply scRNA-seq to profile human and mouse hematopoietic stem and progenitor cells (HSPCs), and infer and validate regulatory networks, in order to understand transcriptional programs in hematopoiesis. We found that regulatory networks were highly conserved between human and mouse, as reflected by the observation that the connectivity of hematopoiesis related genes in co-expression networks built with human and mouse datasets were similar. Regulatory networks were hierarchical in structure and middle level genes were more likely to be related to and collaborative for hematopoietic functions of differentiation and maturation along lineage pathways.

## Methods

### scRNA-seq and data processing

**Fig. 1:**
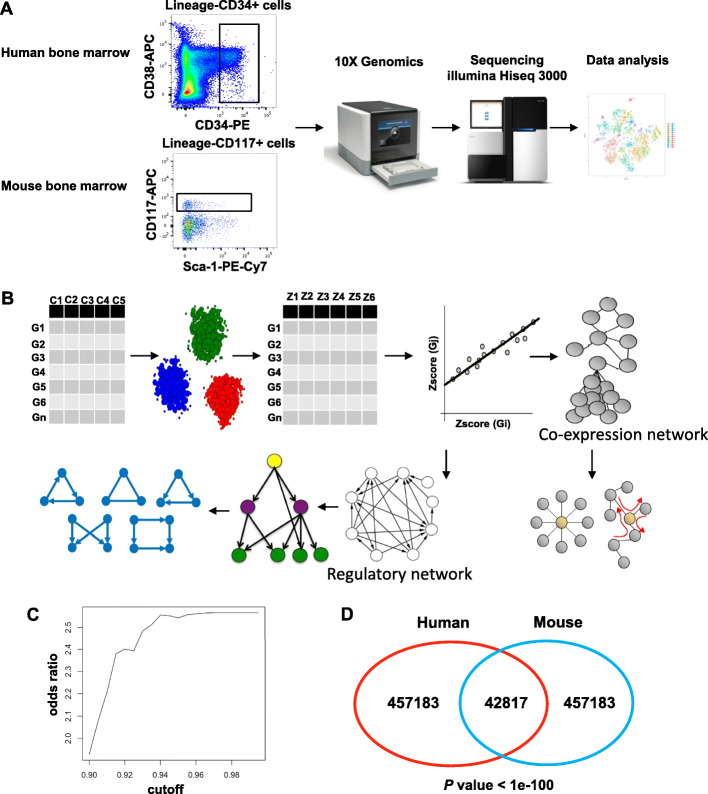
Schematic overview of the study design (**a**) and analysis pipeline (**b**). HSPCs were collected from human and mouse bone marrow, and single cell expression were profiled. Cells were clustered into number of groups and differential expressions (DEs) between pair-wised clusters were calculated. The correlation between pairs of genes was calculated with DEs for co-expression network reconstruction. CLR algorithm was used to correct background and build regulatory networks. **c** Curve of odds ratio for the enrichment of gene pairs in co-expression network in ChIP-chip versus all possible TF-TG pairs with different correlation cutoff. Higher co-expression between TF and target genes identified by ChIP-seq. Higher co-expressed pairs tended to appear more frequently in ChIP-chip validated pairs. **d** Venn diagram showing the overlap of co-expression gene pairs built with human and mouse datasets. Overlap of gene pairs between human and mouse is significantly higher than that expected from random selection (*p* < 1e-100)

Bone marrow samples were obtained from healthy donors after written informed consent in accordance with the Declaration of Helsinki and approved by the institutional review boards of the National Heart, Lung, and Blood Institute. Human bone marrow samples were obtained from healthy donors and processed within 2 h after collection. CD3-CD14-CD19-CD34+ cells were sorted using a LSRII Fortessa Cytometer (BD Biosciences). Lineage-CD117+ cells were sorted from bone marrow of C57BL/6 mice (Fig. 1a). scRNA-seq cDNA libraries were prepared using the Chromium Single Cell ‘3 platform (10 × Genomics). RNA-seq libraries were sequenced with the format of paired-end reads of 75-bp on an Illumina HiSeq 3000 System. Alignment, barcode assignment and Unique Molecular Identifier (UMI) counting were performed using the Cellranger Single-Cell Software Suite. Filtered data only included genes with at least one UMI count detected in at least one cell. Graph-based clusters methods were applied to group cells based on two-dimensional t-distributed Stochastic Neighbor Embedding (tSNE) using Seurat2 at resolution 2 [18]. Each gene from the cluster was compared to the median expression of the same gene from cells in all other clusters [19]. Genes were ranked based on their expression fold change, and the top cluster-specific genes were compared with published cell type-specific genes [20]. An HSPC subtype was assigned to each cluster based on statistical significance of overlap between HSPC- and cluster-specific genes (Fisher’s exact test). Raw data from all experiments have been deposited in the NCBI Gene Expression Omnibus database under the accession numbers GSE135194 and GSE142235.

### Positive control of gene regulations

We downloaded the gene regulation relationships between 60 TFs and target genes (TGs) in the K562 cell line, derived from a patient with a hematologic malignancy, as annotated with ChIP-chip data from the ENCODE project as a positive control [21]. (As there are only 60 TFs profiled in this dataset, its coverage is not complete.)

### Essential genes and cancer-related genes

Lists of essential genes were extracted from a published screen of five human cell lines representing a cross-section of immortalized and cancer tissues [22], to obtain a compilation of 1580 human core fitness genes. Cancer-related genes were downloaded from https://cancer.sanger.ac.uk/census, and genes for blood-related tumor types were retained for our analysis [23]. GWASs data were downloaded from GWAS catalog (https://www.ebi.ac.uk/gwas/) and the susceptibility genes of hematopoiesis were extracted by keeping the traits associated with blood cell.

### Metric for tissue specificity

Tau (*τ*) value proposed by Kryuchkova Mostacci [24] was used as a measure for cell type specificity:

$$ \hat{x_i}=\frac{x_i}{\max (x)},\mathrm{and}\ \tau =\frac{\sum_{i=1}^N\left(1-\hat{x_i}\right)}{N-1}, $$

where *x*_*i*_ is the average expression of each gene of all cells with type *i*. Tau is calculated on the log RNA-seq expression data in this study. The values of *τ* vary from 0 to 1, where 0 means ubiquitous expression, and 1 indicates perfect type specificity. Its advantage for calculating tissue specificity has been validated [24]. In this study, cell type specific genes were defined to be those with *τ* > 0.8, and were assigned to the cell type with the highest expression. We also calculated an average expression value of each gene in each cell type, scaled to Z-scores relative to a mean value across all cell types, and converted to *p* values for cell type specificity, and used it in activated subnetwork extraction.

### Co-expression network reconstruction

We built a human to mouse one-to-one homologous gene list (13,520 genes) collected from InParanoid (http://inparanoid.sbc.su.se) [25]. Co-expression network reconstruction and analysis were performed only for genes in this list. We used bigScale2 (https://github.com/iaconogi/bigSCale2) to build the co-expression networks of human and mouse with datasets GSE135194 and GSE142235, respectively. Co-expression networks are undirected (network edges have no direction).

bigSCales2 uses a recursive strategy to produce the highest number of stable clusters (generated by cell sub-types and subtle cell states), and then conducts an iterative differential expression (DE) analysis between all pairs of clusters. *X* clusters result in a total of *X* ∗ (*X* − 1)/2 unique comparisons and each comparison yields one cluster DE Z-score for each gene that indicates the likelihood of an expression change between the corresponding two clusters. bigSCales2 subsequently computes correlations between genes using DE Z-scores instead of expression values (Fig. 1b).

The distribution of correlations is influenced by biological (disparities between human and mouse) and technical factors (batch effect). To compare networks inferred for the two species, we used an adaptive rather than fixed correlation threshold. Specifically, the co-expression networks were built by retaining the top 500,000 correlations (~ 4%).

### Gene regulatory network reconstruction

The undirected co-expression network was refined by discarding network edges representing pairs of genes in which neither was annotated as “regulator of gene expression”, as we considered such network edges to likely represent spurious co-regulation or other functional association. Transcriptional regulators were defined as those genes with Gene Ontology annotations “nucleic acid binding” or “transcriptional regulation”. We then used the Context Likelihood of Relatedness (CLR)-based corrected Z score approach to assign the regulatory relationship strength to each TF-target pair [26]. The core of CLR is a background correction algorithm that computes the significance of a given correlation *R*_*i*, *j*_ value by comparing it to all R values for gene *i* and all R values for gene *j*. Briefly, let *R* be an *N* × *N* matrix, with each entry, *R*_*i*, *j*_, equals the correlation between a pair of genes (*g*_*i*_, *g*_*j*_) from bigScale2. In order to derive a CLR score for that pair of genes, *z*(*g*_*i*_, *g*_*j*_), first a Z-score for *R*_*i*, *j*_ with respect to the elements in the *i* ’th row of *R* was calculated.

$$ {z}_i\left({g}_i,{g}_j\ \right)=\frac{R_{i,j}-\frac{\sum_k{R}_{i,k}\ }{N}\ }{\sigma_i\ } $$

where *σ*_*i*_ is the standard deviation of the elements in the *i* ’th row of *R*. Second, we computed a Z-score for *R*_*i*, *j*_ with respect to the elements in the *j* ’th column of *R*, i.e.

$$ {z}_j\left({g}_i,{g}_j\ \right)=\frac{R_{i,j}-\frac{\sum_l{R}_{l,j}\ }{N}\ }{\sigma_j\ } $$

where *σ*_*j*_ is the standard deviation of the elements in the *j* ’th column of *R*. Lastly, two Z-scores were combined into a CLR pseudo Z-score, as:

$$ z\left({g}_i,{g}_j\ \right)=\sqrt{z_i{\left({g}_i,{g}_j\ \right)}^2+{z}_j{\left({g}_i,{g}_j\ \right)}^2} $$

After CLR background correction, the *N* × *N* matrix of pseudo Z-scores is used to quantify the confidence of gene-pair relatedness.

$$ Z=\left[\begin{array}{ccc}z\left({g}_1,{g}_1\ \right)& \cdots & z\left({g}_1,{g}_N\ \right)\\ {}\vdots & \ddots & \vdots \\ {}z\left({g}_N,{g}_1\ \right)& \cdots & z\left({g}_N,{g}_N\ \right)\end{array}\right] $$

Then, using the above matrix we retained the confident gene pairs with at least one TF to build a directed network from TF to TG. The R script for network reconstruction was deposited in github (https://github.com/shouguog/hematopoiesis/).

### Identification and functional annotation of conserved and differentially connected genes in co-expression networks

In a gene network, each node *i* represents a gene, and the number of edges attached to a gene, *k*_*i*_, is defined as its connectivity (or degree)

$$ {k}_i= number\ egdes\ with\ gene\ i $$

We defined *k*_*i*, *human*_ and *k*_*i*, *mouse*_ as the connectivity of the homologous genes in the human and mouse networks, respectively. Connectivity values were normalized in respect to network size. To calculate the differential connectivity of gene *i*, we added 10 to each connectivity value in order to reduce the disproportionate fold change among low connectivity genes, and then obtained the difference between human and mouse on a log scale:

$$ Diff{K}_{i, human, mouse}=\mathit{\log}\left({k}_{i, human}+10\right)-\mathit{\log}\left({k}_{i, mouse}+10\right). $$

Genes with values greater than zero were more connected in human, while those with a value less than zero were more connected in mice. A value close to zero (extremely low absolute values of *DiffK*_*i*, *human*, *mouse*_) indicated conserved connectivity between human and mouse.

Gene set enrichment analysis was performed for genes in the two 5% tails, and the middle 10%, of the *DiffK* distribution, using topGO [27]. The top 5% of genes showed higher connectivity in the human network, while the bottom 5% of genes showed higher connectivity in the mouse network. We also defined those with absolute values of differential connectivity in the bottom 10% (the middle 10% of the true value distribution) as the genes with conserved regulation (least differential connectivity).

### Hierarchical organization of GRN

TFs at different levels have different properties [12]. Hierarchical layouts present a more intuitive picture than does the conventional hairball or circular representation. For instance, top-level TFs more strongly influence expression of the whole transcriptome, and middle-level ones tend to transfer information-flow and can be bottlenecks (with high betweenness). We downloaded MATLAB code for the simulated annealing algorithm (http://encodenets.gersteinlab.org), and used it to organize our GRN into a hierarchical structure of four levels. This algorithm arranged all the TFs into different levels in the hierarchy for maximizing the number of edges extending from upper to lower levels [21].

### GRN motif scanning

Network motifs are small structures that occur significantly more frequently than in randomized networks. For every GRN, 1000 randomized networks were generated for reference, by maintaining the in−/out-degree of every network node but rewiring the connected genes randomly. All three- and four-node motifs were identified using mfinder [28], with three criteria: (1) occurrence *>* 5; (2) *p*-value *<* 0.05 and (3) *Z*-score value *>* 2.

### Data and code sharing

Datasets and code are available in GEO repertoire (GSE135194, GSE142235) and github (https://github.com/shouguog/hematopoiesis/tree/master/networkmodeling).

## Results

### Processing of scRNA-seq data of human and mouse

We obtained bone marrow samples from four healthy human donors. In order to characterize the early stages of hematopoiesis, we sorted lineage-CD34+ cells to enrich for HSPCs. In total, after quality control, 15,245 single CD34+ stem/progenitor cells were retained for further analyses.

Sequencing data of single CD34+ cells were visualized in tSNE (Figure S2A). Hematopoietic cell identity was assigned to each cell cluster by comparing cluster-specific genes with a reported lineage signature gene list [29]. CD34+ cells could be computationally assigned to the following subpopulations: multipotent progenitor HSCs, megakaryocyte-erythroid progenitors (MEPs), granulocyte-monocyte progenitors (GMPs), B lymphocyte progenitors (ProBs), and early T lineage progenitors (ETPs) (Figure S2A). The number of clusters identified by Seurat depends on the resolution selected. Although resolutions 1, 2 and 3 generated different numbers of clusters, the cell type assignment was almost identical and did not affect further analyses.

In total, 17,560 Lineage-CD117+ cells from mice were also clustered unsupervised based on transcriptome similarity using tSNE (Figure S2B). Hematopoietic cell identity was assigned to each cluster of cells by comparing cluster-specific genes with accepted lineage signatures [30]. We could group the cells into: long-term hematopoietic stem cells (LTHSC), multipotent progenitors (MPP), lymphoid multipotent progenitors (LMPP), common myeloid progenitors (CMP), megakaryocyte-erythrocyte progenitors (MEP), and granulocyte-monocyte progenitors (GMP) (Figure S2B).

We defined HSC in human, and MPP and LTHSC in mouse as conserved HSC; ProB in human and LMPP in mouse as lymphoid cells; and GMP and MEP in both species as GMP and MEP, respectively.

### Validation of inferred co-expression networks of human and mouse

To compare differences and similarities of the general co-expression landscapes between human and mouse in greater details, we limited our analysis to the 13,520 homologous genes.

We used gene regulation relationships from ChIP-seq as positive controls to assess the inferred networks. We found that TF-target gene (TG) pairs in Chip-chip tended to have higher expression correlation with $odds\ ratio=\frac{\left( fraction\ of\  TF- TG\  pairs> corr\right)}{\left( fraction\ of\ random\ pairs> corr\right)}>2$ at correlation 0.9 and the odds ratio increased with higher correlation (Fig. 1c). Note that our network reconstruction method set a very stringent cutoff to reduce false positives (but risked that true regulatory relationships might be discarded as false negatives, a compromise shared by many algorithms in the field [6]. Additionally, we compared our co-expression results with TF-gene interactions identified in a TF knockdown perturbation experiment [31]. In this experiment, 59 TFs were knocked down individually, and differentially expressed genes were then concluded to be target genes of the knockdown TF. Again significant enrichment odds ratio was evident (Figure S3A). Furthermore, we found that our co-expression networks were supported by known links in public network resources [32–34], including the databases of TRRUS (an expanded reference of TF-TG by literature curation), FunCoup (integrated network) and STRING (integrated protein network). Interacting gene pairs in these datasets all tended to have higher expression correlation (Figure S3B-3G).

### Co-expression conserved between human and mouse

The overlap between human and mouse co-expression gene pairs was much higher than that expected by chance. Specifically, we obtained 42,817 intersected edges between human and mouse co-expression networks (Fig. 1d). In contrast, when we randomly and reiteratively permutated the networks with the same number of nodes and edges, much lower numbers of intersected edges were obtained (8250 ± 72, *p* < 1e-100). Thus, co-expressed gene pairs in human were more likely to be also co-expressed in mouse, and conversely, mouse gene pairs more likely in human, indicating cross-species conservation in gene regulation during hemopoiesis. We termed human-mouse overlapping co-expression gene pairs as the conserved co-expression network. It is reasonable to assume that integration of converged co-expression predictions in both human and mouse improves network reconstruction, and the conserved network may offer a clearer picture of the transcription program during hematopoiesis.

### Co-expression networks showed small world and scale free properties

**Fig. 2:**
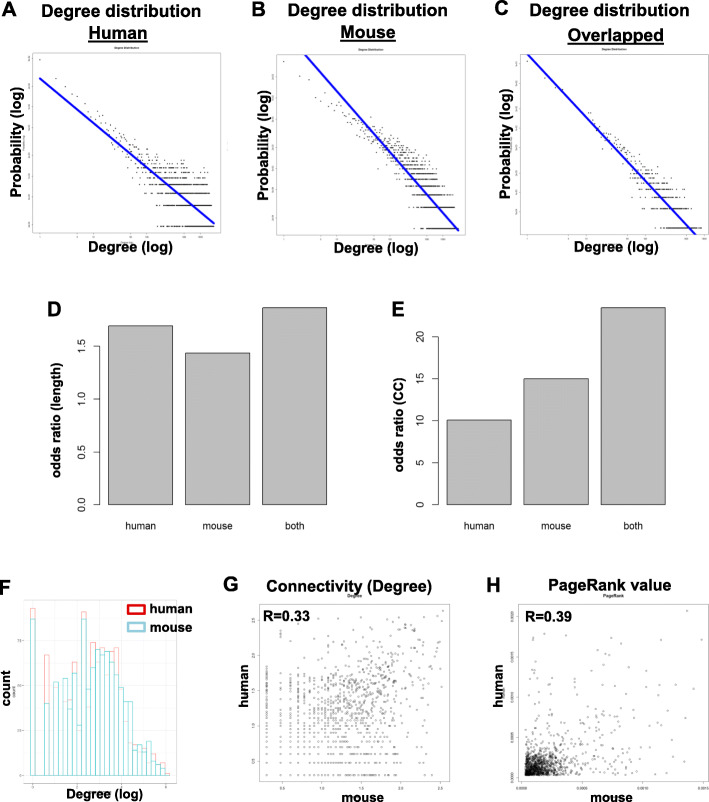
Power-law distribution of gene connectivity in co-expression network of human (**a**), mouse (**b**) and in the conserved network (**c**). **d** Odds ratio of average distance between co-expression network and same-size randomized network in human, mouse and both. **e** Odds ratios of clustering coefficient between co-expression network and same-size randomized network in human, mouse and both. **f** Histogram of log (degree) of genes in human and mouse networks. Correlation in gene connectivity (**g**) and page rank value (**h**) between networks built with mouse and human datasets

Both the individual co-expression networks from human and mouse, and the conserved subnetwork, demonstrated scale-free behavior. Frequency of connectivity showed a negative logarithmic correlation with the connectivity (Fig. 2a-c). Although the average numbers of connections were 377, 282 and 78 respectively, in all three networks most genes were connected to only a few other genes, a hallmark of scale-free networks [21].

We next examined whether co-expression networks were small world by creating randomized networks with the same number of nodes and edges, and comparing the mean shortest path length (L) and the Clustering Coefficient (CC) of networks [35]. The co-expression networks of mouse and human, and the conserved network, had an average shortest path length of 2.81, 3.26 and 4.02, respectively. The shortest path lengths for randomized networks generated with the Erdos model were 1.96 ± 0.05, 1.92 ± 0.06 and 2.15 ± 0.08. The co-expression networks showed CC of 0.597, 0.718 and 0.510, respectively, and randomized networks with the same number of nodes and edges had clustering coefficients 0.04 ± 0.02, 0.07 ± 0.02 and 0.022 ± 0.03, respectively. Thus, the co-expression networks had all the properties of a small-world (L ≈ L random, cc ≫ CC_random_) with some highly connected subnetworks (Fig. 2d-e, Figure S1A).

Our results were robust to modeling at different thresholds other than the top 500,000 correlated gene pairs in yielding similar small-world and scale-free properties (data not shown).

### Conserved and species-specific co-expression connectivity in human and mouse

The range of gene connectivity values in human was generally comparable to that in mouse, suggesting broad structural similarity in gene regulation (Fig. 2f). There was high correlation in genes’ connectivity values between human and mouse networks (Fig. 2g). PageRank confirmed high correlation of human and mouse (Fig. 2h). The DiffK differential connectivity values approximately followed a normal distribution and *p* values were calculated based on normal distribution (Figure S2E). We also calculated FDR with the locfdr package (https://cran.r-project.org/package=locfdr), in which the empirical nulls with parameters were estimated by maximum likelihood. The degree, p value and FDR were shown in Table S1.

To evaluate evolutionary conservation and divergence in network connectivity between human and mouse in more detail, for each gene we calculated a DiffK value of differential connectivity for in silico functional analysis. We ranked homologous genes according to the DiffK differential connectivity values and selected the top and bottom 5% from the list for functional enrichment analysis. The topGO analysis was conducted using functional annotation in the org.hs.eg.db package in Bioconductor (org.mm.eg.db provided similar results; data not shown). The top 1000 over-represented GO terms, both for differentially connected and conserved genes, are shown in Table S1. Genes showing more connection in human or mouse were largely related to general cellular functions, including metabolism and development, such as GO:0035337 (fatty-acyl-CoA metabolic process), GO:0086065 (cell communication involved in cardiac conduction), GO:0030258 (lipid modification), GO:0019395 (fatty acid oxidation), GO:0034440 (lipid oxidation) and GO:0007423 (sensory organ development). In contrast, many functional terms shared by conserved genes were hematopoiesis-related: GO:0002244 (hematopoietic progenitor cell differentiation), GO:0048535 (lymph node development), GO:0045646 (regulation of erythrocyte differentiation), GO:0048821 (erythrocyte development), GO:0043249 (erythrocyte maturation), GO:0030851 (granulocyte differentiation) and GO:0002714 (positive regulation of B cell mediated immunity). These results suggest species conservation is able to improve gene network reconstruction, consistent with evolutionary selection theory. Genes with strongly conserved connectivity were generally to be functionally evolutionary stable, and play important roles in hematopoiesis [36].

### Gene regulatory networks identify essential and hematopoietic cell-type specific genes

**Fig. 3:**
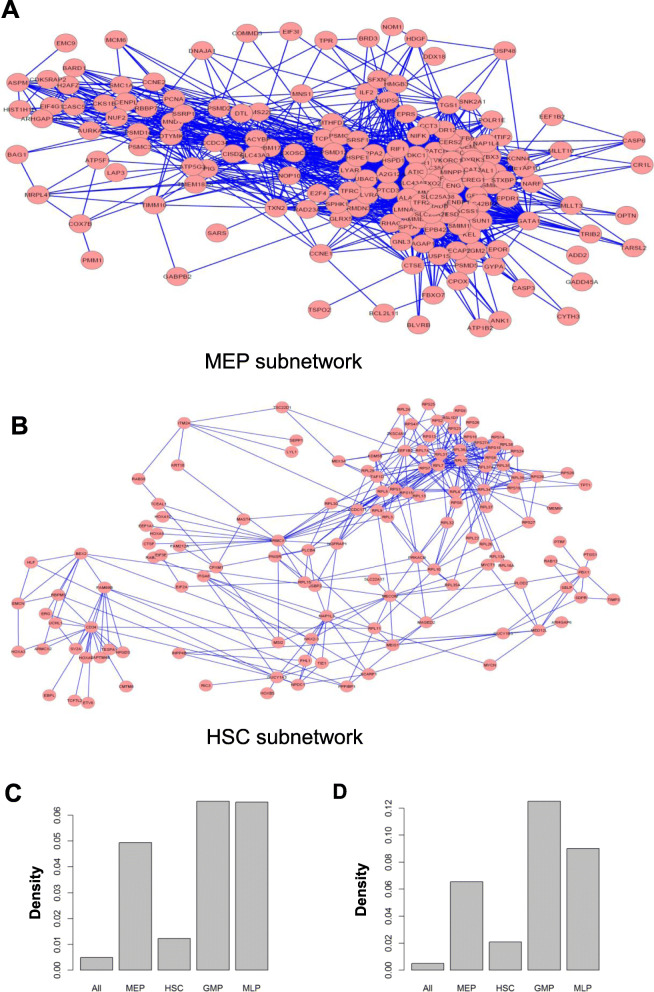
**a** Top active co-expression subnetwork expressed in MEP progenitors identified by jActiveModules. **b** Top active co-expression subnetwork expressed in HSC progenitors identified by jActiveModules. **c-d** Network density of co-expression networks built with two different thresholds (top 500,000 and 1,000,000)

Next we examined type-specific transcription programs in different cell types. For this purpose, a Cytoscape plug-in, jActiveModules, was utilized to identify cell type-specific subnetworks (connected sets of genes with unexpectedly high levels of expression in certain cell types) in conserved networks [37]. A cell type-specific subnetwork might include genes that are not specifically expressed but are tightly connected to other highly specific genes. We chose jActivemodules because it can integrate multiple *p* values, and thus utilize gene specificity values tau (*p values for τ*) of both human and mouse. Subnetworks are shown in Fig. 3a-b and Figure S2C-D.

The ontological annotations of genes in cell type-specific subnetworks agreed with their functionality. Table S2 shows the top 1000 GO biological processes terms that were significantly enriched and their corresponding functions in different subnetworks of hematopoiesis. With microarray (human bulk sample) data, Noa Novershtern et al. identified lineage specific modules of highly co-expressed genes during hematopoiesis [38]. The genes in their modules were observed to be highly co-expressed also in our study with an average R value of 0.55.

HSC-specific subnetworks were present for ribosomal protein genes or genes that function mainly in cell differentiation and stem cell proliferation. The main terms included: GO:0008283 (cell proliferation); GO:0035239 (tube morphogenesis); GO:0072089 (stem cell proliferation) and GO:0042254 (ribosome biogenesis).

Genes in the MEP subnetwork mainly functioned in erythropoiesis. The main terms included: GO:0048821 (erythrocyte development); GO:0002262 (myeloid cell homeostasis); GO:0030218 (erythrocyte differentiation) and GO:0034101 (erythrocyte homeostasis).

Genes in the GMP subnetwork (Figure S2D) mainly functioned in the immune system. The main terms included: GO:0006955 (immune response); GO:0002446 (neutrophil mediated immunity); GO:0036230 (granulocyte activation); GO:0042119 (neutrophil activation); GO:0002252 (immune effector process); GO:0002444 (myeloid leukocyte mediated immunity); GO:0002275 (myeloid cell activation involved in immune response) and GO:0002263 (cell activation involved in immune response).

Genes in the LMPP/ProB (lymphoid cells) subnetwork (Figure S2C) also were immune-related. The main terms included: GO:0030217 (T cell differentiation); GO:0045321 (leukocyte activation); GO:0030098 (lymphocyte differentiation); GO:0001775 (cell activation); GO:0042110 (T cell activation); GO:0036037 (CD8-positive); GO:0006955 (immune response); GO:0002521 (leukocyte differentiation) and GO:0048534 (hematopoietic or lymphoid organ development).

Generally speaking, topological features of genes in a network associate to their biological importance [21]. Genes with high connectivity are termed “hub genes” and are usually functionally important. “Betweenness” measures the number of the shortest paths transiting through the gene, and highest betweenness genes control most of the information flow in the network, representing the critical nodes of the network (such as G11 in Figure S1A). Betweenness is a better indicator of essentiality than is gene connectivity, although they are usually highly correlated. Network connectivity and betweenness of gene lists are shown in Supplemental File 1. Some individual examples are provided below.

In normal hematopoiesis, MEIS1 expression is correlated with cell self-renewal, with levels highest in HSCs and declining with differentiation. In mouse, *MEIS1* is required to maintain functional LT-HSCs [39], and *MEIS1* was indeed the top hub gene in the HSC subnetwork.

*GATA1* is a hub gene with high betweenness in the MEP subnetwork. *GATA1* is expressed in primitive and definitive erythroid cells and megakaryocytes, and gene-targeting studies have confirmed its importance in these cells. For examples, in chimeric mice, *GATA1*-null erythroid cells fail to mature beyond the proerythroblast stage, and absence of *GATA1* in megakaryocytes leads to increased proliferation and deficient maturation of megakaryocytic progenitors [40].

*CD48* is a hub gene with high betweenness in the LMPP/ProB subnetwork. This gene encodes a member of the CD2 subfamily of immunoglobulin-like receptors which appear on the surface of lymphocytes and other immune cells, and participate in activation and differentiation pathways in these cells. Cd48−/− mice are severely impaired in CD4+ T cell activation on signaling through the T cell receptor [41].

### HSC subnetwork was sparse in comparison to differentiated cell subnetworks

When network density, the ratio of edge number to the total number of all possible edges (2 ∗  # *edge*/ # *node*/(#*node* − 1) ), was calculated, the HSC subnetwork showed the lowest density. Low density indicated functional diversity of expressed genes and broad differentiation potential at this stage. Genes driving differentiation toward distinct lineages were active in the HSC subnetwork, but they did not display the high co-expression characteristic as in cells fully committed to differentiation stages. In differentiated cells, only functionally related genes specific to the cell type were highly expressed and cooperated to form a denser co-expression network (Fig. 3c-d). The co-expression network in its entirety showed low density due to the functional diversity of genes expressed.

**Fig. 4:**
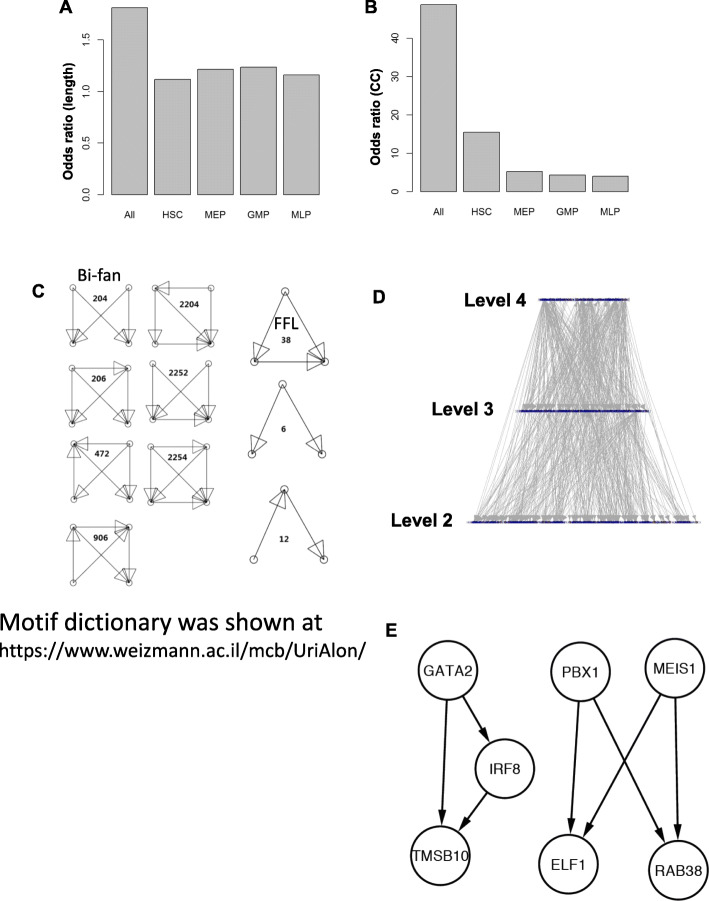
**a**. Odds ratios of average distance between genes (i.e. length of shortest path) in the co-expression network versus that in the same size randomized network. **b** Odds ratios of clustering coefficient between the co-expression network and the same-size randomized network. **c** Four node motifs observed in co-expression networks and tissue specific sub-networks. The ID can be found in Supplemental file S3 and https://www.weizmann.ac.il/mcb/UriAlon/sites/mcb.UriAlon/files/uploads/NetworkMotifsSW/mfinder/motifdictionary.pdf. **d** Hierarchical structure of GRN (level 1 genes were excluded due to large number of genes). There were lower numbers of TFs in higher levels of hierarchy. **e** Example FFL and bi-fan motifs identified in hematopoietic GRN

All subnetworks showed small world properties when compared to randomized networks. The whole network, HSC, MEP, GMP and LMPP/ProB specific subnetworks had the average shortest path lengths of 5.2, 3.9, 2.4, 2.4 and 2.3, respectively. In contrast, Erdos random networks containing the same number of nodes and edges had the average shortest path lengths of 2.86 ± 0.06, 3.54 ± 0.08, 1.96 ± 0.09, 1.95 ± 0.06 and 1.97 ± 010, respectively. The whole network, HSC, MEP, GMP and LMPP/ProB subnetworks had clustering coefficients of 0.49, 0.33, 0.51, 0.57 and 0.499, respectively. In contrast, the clustering coefficients of Erdos random networks with the same number of nodes and edges were much lower at 0.01 ± 0.02, 0.021 ± 0.03, 0.097 ± 0.04, 0.13 ± 0.03 and 0.123 ± 0.04, respectively. Again, the co-expression subnetworks showed small-world properties (L≈ > L_random_, cc ≫ cc_random_). The clustering coefficient in HSC had the highest odds value versus randomized network, as the HSC subnetwork showed the most distinct small world properties (Fig. 4a-b).

When the subnetworks were created with the top 1000 tau values of all cell types, rather than constructed using the jActiveModules algorithm, similar results were obtained (data not shown).

### Gene regulatory network reconstruction and analysis

We combined the advantages of a context-based method [26] and Z-score based bigScale2 approaches. Correlations were calculated with transformed variables, in which expression counts were replaced by Z-scores derived from cell clusters. The correlation matrix was then scaled by row and column to identify significant gene pairs, and gene ontology (GO) information was used to trim the network to “regulators of gene expression” in order to retain only putative causal relationships. We obtained a GRN with 469 genes and 1017 edges with threshold of 5; and 2570 genes and 9060 edges with threshold of 3. The final regulatory hierarchy had > 85% of its edges directed downward. For co-expression networks, we identified the subnetwork of GRNs which was specifically expressed in different cell types with jActiveModules (results not shown).

### Network motifs in GRNs

Motifs are defined as the small building blocks in networks, for which appearance frequency is significantly higher than the expected value from randomized networks. Motifs are considered the smallest structural and functional units of a network. In GRN, motifs are typically composed of several TFs that co-regulate in characteristic patterns, and their target genes, as for examples, bi-fans, FFL, or negative feedback loops [21]. We identified all 3- and 4-node sub-graphs (see Fig. 4c and Supplemental file S3) in the GRN and compared their occurrence in randomized networks using the sampling tool mfinder to identify motifs [28]. We distinguished motifs shared by GRNs and all cell type-specific subGRNs, from those that only occurred in a few cell types, and denoting the latter cell type-specific motifs (CTSM). There were two shared and eight CTSM motifs. FFL and bi-fan motifs were observed in both GRN and all the cell type specific subGRNs. Thus, combinatory regulation at the transcriptional stage tended to be topologically similar across different cell types.

FFL is a motif in GRNs of many organisms (yeast, *E. coli*, mouse, and human). FFL is defined as a motif with three nodes: two TFs (B3 and C4) and a target gene (D4), in which B3 regulates C4, and both jointly regulate D4 (Figure S1B). Both experimental data and computational simulations suggest that FFL serves as a sensitive delay element: expression of a target gene is delayed due to the required stimuli from both TF1 (B3) and TF2 (C4), and that time is required awaiting the signal from TF1 for TF2 (C4) to accumulate to a critical level to activate transcription of D4. FFL filters fluctuations from short transient stimuli (of insufficient duration or strength to allow C4 accumulation to a critical value needed to activate D4). Further combination of FFLs can generate multi-output FFLs with different functions, such as generating “first in, first out” patterns in the transcripts of target genes [42]. Bi-fan motifs are statistically over-represented in a gene regulatory network: here, two TFs co-regulate two target genes. Bi-fan motifs appear to temporally regulate signal propagation and to synchronize outputs from the two TFs, analogous to OR gate or AND gate in electronic circuits [43]. One FFL and one bi-fan motifs were shown in Fig. 4e. All three genes in FFL (GATA2, IRF8, TMSB10) were correlated with haematological characteristics. Four genes in bi-fan (MEIS1, RAB38, PBX1, ELF1) were associated with hematopoietic stem cell.

### Pyramidal regulatory hierarchies in networks

Hierarchical layout of a GRN provides a more intuitive picture than does the conventional hairball or circular presentation. Previous work has revealed that TFs at different levels often have different properties. For instance, in E coli and yeast, top-level TFs more strongly influence expression of the whole transcriptome, and middle-level TFs tend to transfer information flow and can be bottlenecks (with high betweenness) [12].

**Fig. 5:**
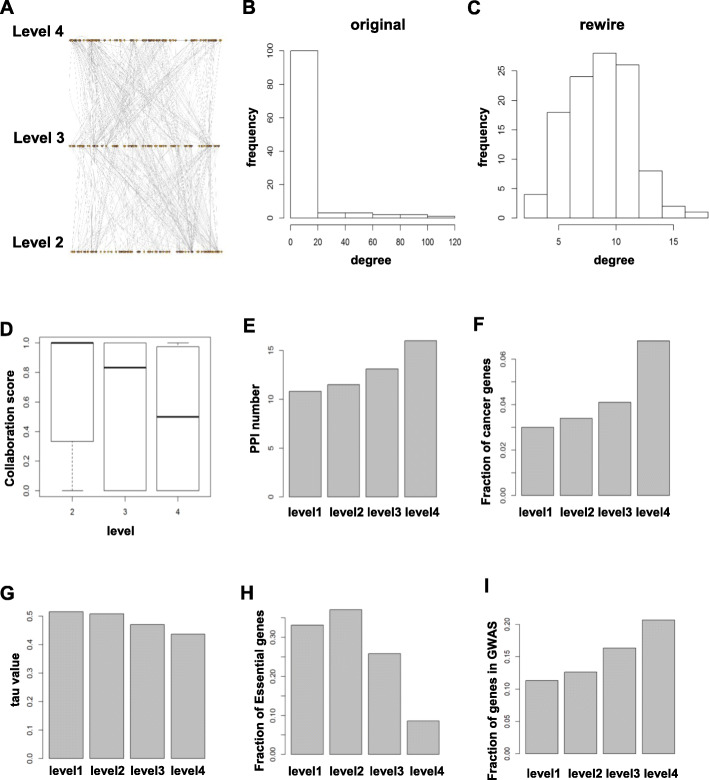
**a** Hierarchical structure of randomized GRNs. The level 1 genes were excluded. **b** The histogram of degree (connectivity) of genes in the GRN, which showed power-law distribution. **c** The histogram of degree (connectivity) of genes in the random GRN, in which power-law distribution was lost. **d** Collaboration scores of genes at different levels. **e** Average numbers of interacting proteins of genes at different levels of the GRN hierarchy. **f** Fraction of cancer related genes at different levels. TFs at higher levels of the hierarchy were more associated with cancer (**g**) tau (cell type specificity) scores of genes at different levels. TFs at the bottom level of the hierarchy were cell type specifically expressed in hematopoiesis. **h** Fraction of essential genes at different levels. TFs at the bottom level of the hierarchy had a strong tendency to be essential genes. **i** Fraction of hematopoiesis related genes as annotated by GWAs at different levels. TFs at higher levels of the hierarchy were more associated with hematopoietic traits in GWAS studies

The hematopoietic regulatory network had a four-layer pyramid-shaped hierarchical structure (Fig. 4d): the number of TFs on each level was smaller than that of the previous level (a similar pyramidal hierarchy has been observed in *E. coli* and yeast). We investigated randomized networks for similar hierarchical organizations. After randomly rewiring the edges between TFs and their targets within the regulatory network as a whole, the pyramid-shaped hierarchical structure disappeared (Fig. 5a). Furthermore, the average out-degrees of all TFs were equal in randomized networks, markedly distinct from the established GRN (Fig. 5b-c). Similar results were obtained using this approach when randomly rewiring yeast GRNs [12].

About 20 % of genes located to the top level (level 4), where there was only a small number of TFs (Fig. 4d). Most TFs had partners to co-regulate other genes, rather than acting alone, a characteristic of “democratic” rather than “autocratic” hierarchies [44].

### Functional properties of TFs at different levels of the GRN hierarchy

After functional annotations of all TFs at different levels (Table S3), we discovered that top-level TFs tended to regulate gene expression and differentiation. Terms included: GO:0006355 (regulation of transcription) GO:0010467 (gene expression), GO:0030154 (cell differentiation) and GO:0032502 (developmental process).

In contrast, TFs in middle levels (level 2–3) were associated with pathways related to hematopoiesis. Main functional terms for the level 3 included: GO:0061515 (myeloid cell development), GO:0009888 (tissue development), GO:0030218 (erythrocyte differentiation), GO:0034101 (erythrocyte homeostasis) and GO:0045165 (cell fate commitment). Main functional terms for the level 2 included: GO:0036230 (granulocyte activation), GO:0002275 (myeloid cell activation involved in immune response) GO:0040008 (regulation of growth), GO:0043299 (leukocyte degranulation) and GO:0002444 (myeloid leukocyte mediated immunity).

Genes locating at the bottom level were responsible to general functions such as GO:0007049 (cell cycle) and GO:0006281 (DNA repair).

Overall, top level TFs functioned in general pathways of cell development and differentiation, and those at the middle level defined cell type-specificity.

For each TF, we defined a collaboration score [(#*of target genes which are cotargeted by other TFs*)/(#*of target genes*)] as the ratio of coregulated to total target genes (TF C1 in Figure S1B). Level 2 members had collaboration scores at the maximum value of 1: almost all TFs had at least one coregulation partner for every target gene they regulated (Fig. 5c-d). Such a pattern of gene regulations in a GRN that is highly collaborative because TFs partner with other TFs and very few act alone to regulate downstream target genes. TFs at the lower level had higher collaboration scores (Fig. 5d). TFs at the higher levels of the hierarchy mainly regulate genes engaged in general cellular functions., and they require fewer interactions due to high tolerance for fluctuations in expression. In contrast, TFs at lower levels of the hierarchy must collaborate for appropriate expression of cell type-specific transcription programs [44]. Genes targeted by the same TFs have similar functions and are co-expressed, with the degree of co-expression increasing as the number of shared TFs increases [45].

We examined tau (*τ*) values of cell-type specificity of genes at different levels, and found that the genes at the middle level were more likely to be selectively expressed in certain cell types than those at the top level. This result was consistent with the functional analysis described above and further supported the inference that differentiation was mainly controlled by middle level TFs (Fig. 5g).

### External signals to the GRN

In a hierarchical GRN, regulation of gene expression in cells normally occurs in a multistep fashion, starting from TFs at the top of the hierarchy, which receive stimulating signals from outside and have more external partners. The roles of top-level TFs in receiving and processing external signals can manifest in higher number of interactions with other proteins (directly or indirectly via receptors). Indeed, in our analysis, top-level TFs on average showed more interactions with other proteins than did TFs of other levels, as was observed in yeast and *E. coli* [12] (Fig. 5e).

### GRN hierarchy and organism survival and disease

TFs at higher levels can indirectly regulate genes through intermediate TFs, and thus higher-level TFs are more influential in GRN. The importance of these TFs to the GRN hierarchy would also indicate that aberrations in them are more likely to cause the organism to deviate from normal healthy physiology toward a disease process. We investigated the role of TFs in initiating disease, especially blood related cancers. Results are displayed in Fig. 5f. Evidently, human TFs at higher levels were more likely to be blood cancer-related, confirming the power of influence of high-level TFs in the hierarchy. Similarly, high level genes tend to appear in the reported gene lists associated with hematopoietic traits collected in GWAS category (Fig. 5i).

TFs at higher levels are more influential, and they regulate gene expression through the transcription cascades. In GRN, top-level TFs might be expected to be essential [22]. However, in our reconstructed GRN, TFs at the lower levels of the network tended to be essential genes, more than those at higher levels (Fig. 5h), as has been observed in GRNs of yeast and *E. coli* [12]. TFs at the top of the transcription regulation hierarchy coordinate gene expression across different pathways, which remain functional upon deletion of these TFs, even though the qualitative expression pattern and quantitative levels of expression in these pathways may be altered. TFs at the bottom of the hierarchy regulate critical biological processes, and their disruption is not tolerated by the cell [12].

## Conclusion and discussion

Our comparative transcriptomic analysis of the hematopoietic system revealed evolutionary conservation in hematopoietic gene regulation across human and mouse. Genes in the conserved co-expression networks were more related to hematopoiesis than those in networks built from human or mouse only.

In this study, we carried out comparative transcriptomic analysis of the hematopoietic systems in human and mouse, through co-expression network and regulatory network modeling. Co-expression networks that were well conserved between these two disparate animals were more related to hematopoiesis than networks built with the data of human or mouse only. In addition, the HSC subnetwork had distinct network features compared to differentiated cell subnetworks, such as lower network density and higher clustering coefficients. Mouse modelling is a powerful tool to validate functions of homologous genes in humans. Pathophysiology can be brought by abnormalities of interactions among gene modules, and inference or validation of functions of network perturbation in human disease in mouse models heavily depends on the evolutionary conservation of networks between mouse and human [46]. Our current analysis only encompassed human and mouse, and addition of data from more species, when they become available, would be helpful in assessing the evolution of hematopoiesis.

Genes most evolutionarily conserved in connectivity tended to be specific to a biological process, hematopoiesis in the current study, thus providing clues for gene functional study with the mouse model. The HSC subnetwork had particular network features compared to differentiated cell subnetworks, such as lower network density and higher clustering coefficients. Similar to GRNs in yeast and *E. coli*, we found that the GRNs in human and mouse hematopoietic systems also possessed a pyramidal hierarchical structure. Decision making in a hierarchy structure is a multi-level process, in which information is transferred from top to bottom. TFs at the top receive outside signals and regulate more genes, and TFs at the middle levels are responsible for regulating and coordinating the genes at the bottom level for lineage specific functions. Further, GR during hematopoiesis also utilizes FFL and bi-fan to organize the regulation program, same as GRNs in other organisms during other biological processes. GRN level-specific functions at different levels and network motif sharing indicate evolutionary conservation at the level of organization and hierarchy of GRNs. Although the yeast GRN was built from genetic and ChIP-chip experiments, and our GRN was imputed from single cell gene expression data, they shared similar pyramid hierarchy and network motifs. In addition to conservation across species, this concordance supports the robustness of our network reconstruction algorithms.

Our study only used gene expression data. However, including other types of experimental data and resources would be valuable, as in incorporating and integrating genomic expression, genome sequences, proteomic data, protein-DNA binding data, high-content gene association inferred from molecular pathology and text mining of the published literature. Diverse data sources can be integrated in parallel or with Bayesian approaches [3]. Beyond protein coding genes, miRNA and lncRNA data would add yet more layers to the depth of understanding of gene regulation [47]. Further, our datasets were derived only from stem and progenitor cells, and comparison with the transcription network in differentiating and mature blood cells may enlarge the perspective of hematopoiesis.

There are many challenges in the computational reconstruction of gene networks. Both metrics to quantify and criteria to define gene regulation relationships will need to be optimized. Novel experimental developments, different methods of dataset integration, and more and efficient algorithms will improve the accuracy of gene network descriptions.

## Supplementary Information

**Additional file 1: Figure S1.** (A) Illustration of network essentiality measures and the small world property. (B) Illustration of hierarchical layers and network motifs. bi-fan motif includes four genes (A1, A2, B1 and B2), and is colored with dark green. FFL motif includes three genes (B3, C4 and D4), and is colored with pink. **Figure S2.** tSNE plots of human (A) and mouse (B) hematopoietic cells, colored by cell types (C) The co-expression subnetwork expressed in lymphoid progenitors (D) The co-expression subnetwork expressed in GMP progenitors **Figure S3.** (A) Curve of odds ratio for the enrichment of gene pairs in co-expression network in TF perturbation experiment versus all possible TF-TG pairs with different correlation cutoff. (B-G) Curves of odds ratio for the enrichment of gene pairs in co-expression network in annotation in STRING (B-C), FunCorp (D-E) and TTRUST (F-G) versus all possible TF-TG pairs with different correlation cutoff

**Additional file 2: Table S1.** Gene Ontology enrichment analysis results of genes in human, mouse and conserved networks. Degree of homologous genes in networks of human and mouse

**Additional file 3: Table S2.** Gene Ontology enrichment analysis results of genes in cell type specific subnetworks

**Additional file 4: Table S3.** Gene Ontology enrichment analysis results of TFs at different levels

**Additional file 5: Supplemental file 1**. Degree and betweenness of gene lists in cell type specific networks

**Additional file 6: Supplemental file 2.** The gene regulatory network with threshold of 3

**Additional file 7: Supplemental file 3.** 3-node and 4-node motifs identified by mfinder in GRN and cell type specific subGRNs

## Data Availability

The datasets generated and analysed during the current study are available in the GEO repository with accession number GSE135194 and GSE142235 (https://www.ncbi.nlm.nih.gov/geo/query/acc.cgi?acc=GSE142235, https://www.ncbi.nlm.nih.gov/geo/query/acc.cgi?acc=GSE135194).

## Abbreviations

HSC: Hematopoietic Stem Cell
HSPC: Hematopoietic Stem and Progenitor Cell
tSNE: t-distributed Stochastic Neighbor Embedding
DE: Differential expression
CC: Clustering Coefficient
GO: Gene ontology
TF: Transcription factor
TG: Target gene
GRN: Gene Regulatory Network
CLR: Context Likelihood of Relatedness
FFL: Feed-forward loop
MAGIC: Markov Affinity-based Graph Imputation of Cells
GWAS: Genome-wide association study

## References

[1] Goode DK (2016). Dynamic gene regulatory networks drive hematopoietic specification and differentiation. Dev Cell.

[2] Zhao X (2017). Single-cell RNA-seq reveals a distinct transcriptome signature of aneuploid hematopoietic cells. Blood.

[3] Gao S, Wang X (2011). Quantitative utilization of prior biological knowledge in the Bayesian network modeling of gene expression data. BMC Bioinformatics.

[4] He F, Balling R, Zeng AP (2009). Reverse engineering and verification of gene networks: principles, assumptions, and limitations of present methods and future perspectives. J Biotechnol.

[5] Hamey FK (2017). Reconstructing blood stem cell regulatory network models from single-cell molecular profiles. Proc Natl Acad Sci U S A.

[6] Chen S, Mar JC (2018). Evaluating methods of inferring gene regulatory networks highlights their lack of performance for single cell gene expression data. BMC Bioinformatics.

[7] Wang J (2016). Single-cell co-expression analysis reveals distinct functional modules, co-regulation mechanisms and clinical outcomes. PLoS Comput Biol.

[8] Wu AR (2014). Quantitative assessment of single-cell RNA-sequencing methods. Nat Methods.

[9] Iacono G, Massoni-Badosa R, Heyn H (2019). Single-cell transcriptomics unveils gene regulatory network plasticity. Genome Biol.

[10] van Dijk D (2018). Recovering gene interactions from single-cell data using data diffusion. Cell.

[11] Erwin DH, Davidson EH (2009). The evolution of hierarchical gene regulatory networks. Nat Rev Genet.

[12] Yu H, Gerstein M (2006). Genomic analysis of the hierarchical structure of regulatory networks. Proc Natl Acad Sci U S A.

[13] Song L, et al. A transcription factor hierarchy defines an environmental stress response network. Science. 2016;354(6312). 10.1126/science.aag1550 PMC5217750 27811239

[14] Cauwels A, Vandendriessche B, Brouckaert P (2013). Of mice, men, and inflammation. Proc Natl Acad Sci U S A.

[15] Fruhmann G (2017). Yeast buddies helping to unravel the complexity of neurodegenerative disorders. Mech Ageing Dev.

[16] Gurumurthy CB, Lloyd KCK. Generating mouse models for biomedical research: technological advances. Dis Model Mech. 2019;12(1). 10.1242/dmm.029462 PMC6361157 30626588

[17] Lai S (2018). Comparative transcriptomic analysis of hematopoietic system between human and mouse by microwell-seq. Cell Discov.

[18] Satija R (2015). Spatial reconstruction of single-cell gene expression data. Nat Biotechnol.

[19] Zheng GX (2017). Massively parallel digital transcriptional profiling of single cells. Nat Commun.

[20] Guo M (2015). SINCERA: a pipeline for single-cell RNA-Seq profiling analysis. PLoS Comput Biol.

[21] Gerstein MB (2012). Architecture of the human regulatory network derived from ENCODE data. Nature.

[22] Hart T (2015). High-resolution CRISPR screens reveal fitness genes and genotype-specific cancer liabilities. Cell.

[23] Futreal PA (2004). A census of human cancer genes. Nat Rev Cancer.

[24] Kryuchkova-Mostacci N, Robinson-Rechavi M (2017). A benchmark of gene expression tissue-specificity metrics. Brief Bioinform.

[25] Sonnhammer EL, Ostlund G (2015). InParanoid 8: orthology analysis between 273 proteomes, mostly eukaryotic. Nucleic Acids Res.

[26] McKinney-Freeman S (2012). The transcriptional landscape of hematopoietic stem cell ontogeny. Cell Stem Cell.

[27] Alexa A, Rahnenfuhrer J, Lengauer T (2006). Improved scoring of functional groups from gene expression data by decorrelating GO graph structure. Bioinformatics.

[28] Kashtan N (2004). Efficient sampling algorithm for estimating subgraph concentrations and detecting network motifs. Bioinformatics.

[29] Laurenti E (2013). The transcriptional architecture of early human hematopoiesis identifies multilevel control of lymphoid commitment. Nat Immunol.

[30] Nestorowa S (2016). A single-cell resolution map of mouse hematopoietic stem and progenitor cell differentiation. Blood.

[31] Cusanovich DA (2014). The functional consequences of variation in transcription factor binding. PLoS Genet.

[32] Han H (2018). TRRUST v2: an expanded reference database of human and mouse transcriptional regulatory interactions. Nucleic Acids Res.

[33] Ogris C, Guala D, Sonnhammer ELL (2018). FunCoup 4: new species, data, and visualization. Nucleic Acids Res.

[34] Szklarczyk D (2019). STRING v11: protein-protein association networks with increased coverage, supporting functional discovery in genome-wide experimental datasets. Nucleic Acids Res.

[35] Watts DJ, Strogatz SH (1998). Collective dynamics of 'small-world' networks. Nature.

[36] Ideker T, Krogan NJ (2012). Differential network biology. Mol Syst Biol.

[37] Ideker T (2002). Discovering regulatory and signalling circuits in molecular interaction networks. Bioinformatics.

[38] Novershtern N (2011). Densely interconnected transcriptional circuits control cell states in human hematopoiesis. Cell.

[39] Kocabas F (2012). Meis1 regulates the metabolic phenotype and oxidant defense of hematopoietic stem cells. Blood.

[40] Ferreira R (2005). GATA1 function, a paradigm for transcription factors in hematopoiesis. Mol Cell Biol.

[41] Gonzalez-Cabrero J (1999). CD48-deficient mice have a pronounced defect in CD4(+) T cell activation. Proc Natl Acad Sci U S A.

[42] Kurata H (2014). BioFNet: biological functional network database for analysis and synthesis of biological systems. Brief Bioinform.

[43] Lipshtat A (2008). Functions of bifans in context of multiple regulatory motifs in signaling networks. Biophys J.

[44] Bhardwaj N, Yan KK, Gerstein MB (2010). Analysis of diverse regulatory networks in a hierarchical context shows consistent tendencies for collaboration in the middle levels. Proc Natl Acad Sci U S A.

[45] Yu H (2003). Genomic analysis of gene expression relationships in transcriptional regulatory networks. Trends Genet.

[46] Barabasi AL, Gulbahce N, Loscalzo J (2011). Network medicine: a network-based approach to human disease. Nat Rev Genet.

[47] Wu Z (2019). Long noncoding RNAs of single hematopoietic stem and progenitor cells in healthy and dysplastic human bone marrow. Haematologica.

