# Supplementary material for: Comprehensive network modeling from single cell RNA sequencing of human and mouse reveals well conserved transcription regulation of hematopoiesis
Source: BMC Genomics. 2020 Dec 29;21(Suppl 11):849. doi: 10.1186/s12864-020-07241-2 (PMC7771096; doi:10.1186/s12864-020-07241-2)

A

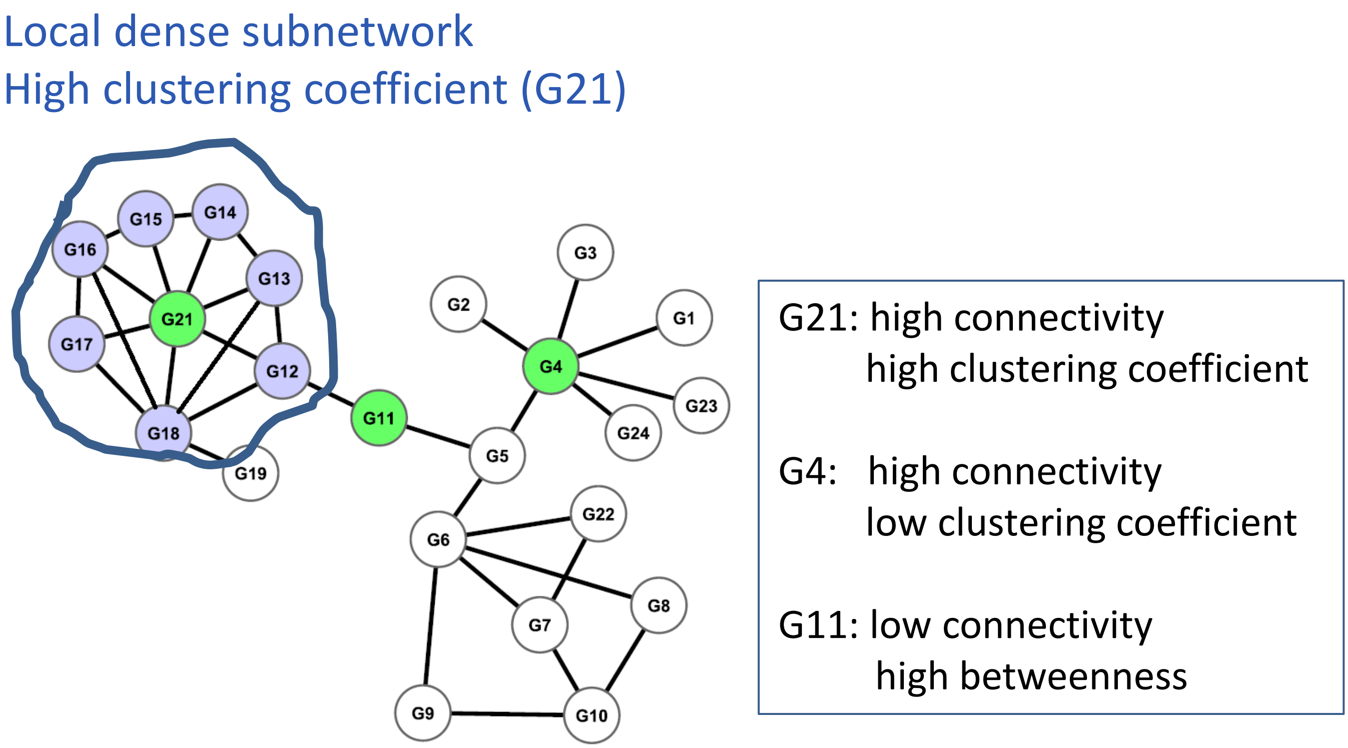

B

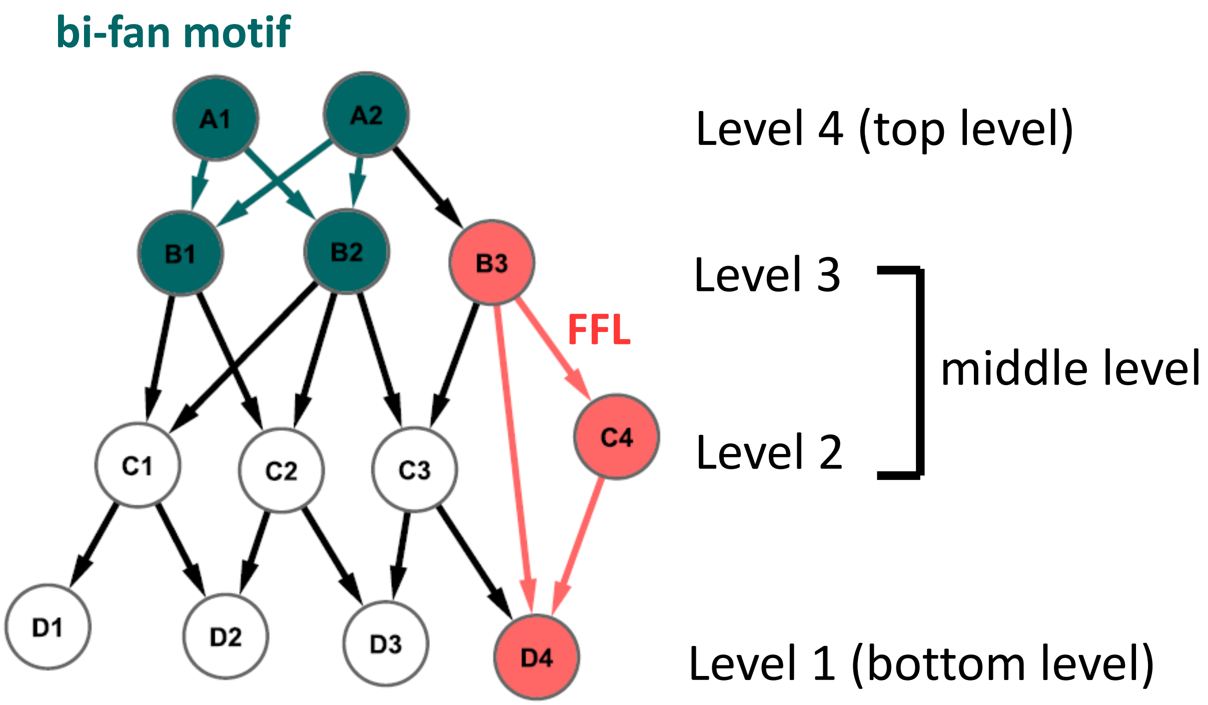

C1 regulates two genes (D1, D2), and D2 is co-regulated. Its collaboration score is ½=0.5.

Gao et al., Figure S2

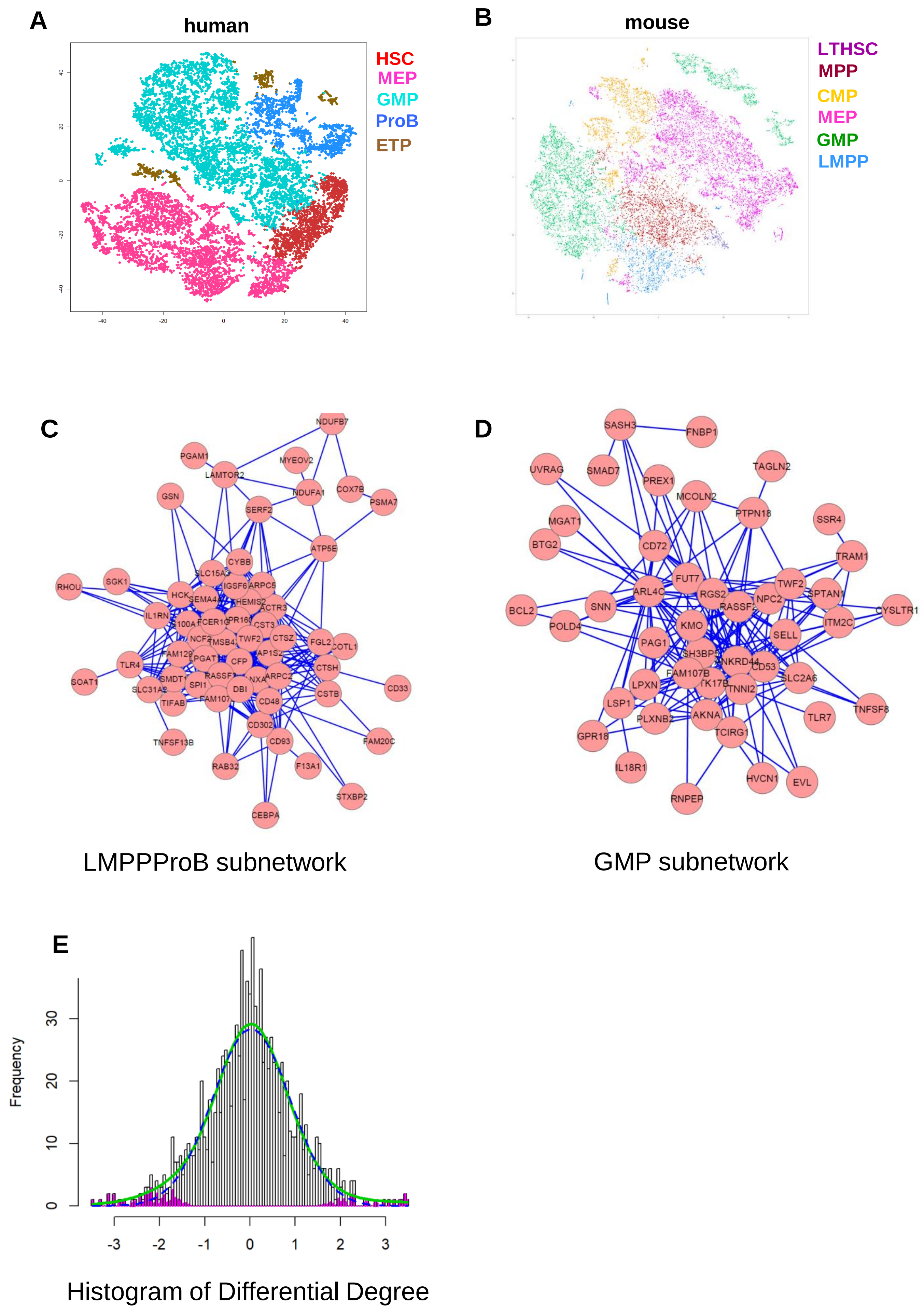

Gao et al., Figure S3

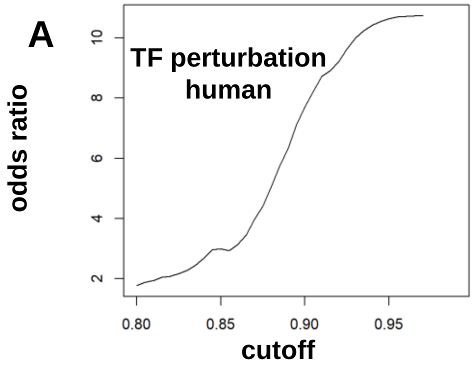

$$odds\ ratio = \frac{(fraction\ of\ \text{gene}\ pairs > corr)}{(fraction\ of\ random\ \ pairs > corr)}$$

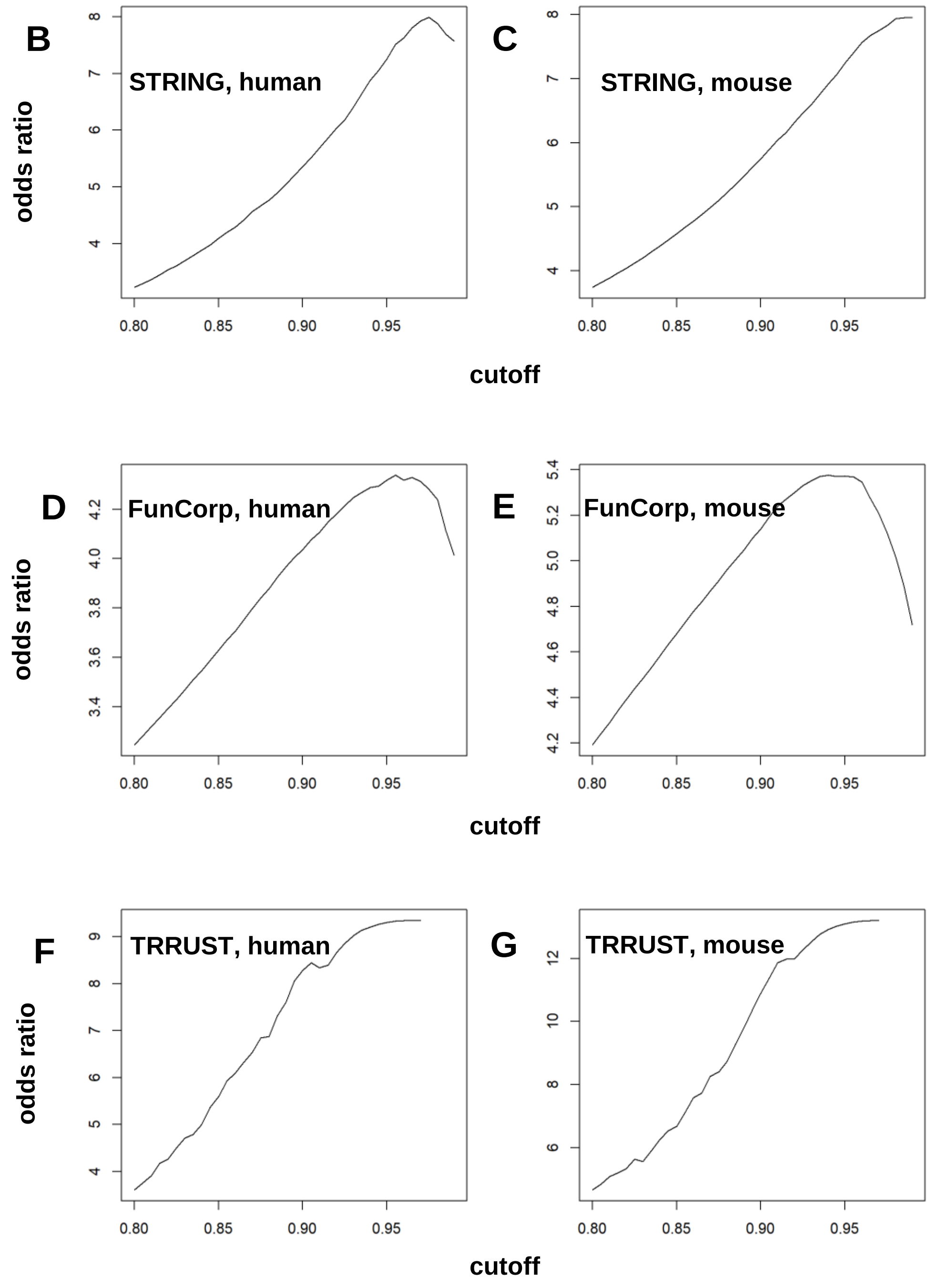

Supplement: Supplementary file 1 — Additional file 1: Figure S1. (A) Illustration of network essentiality measures and the small world property. (B) Illustration of hierarchical layers and network motifs. bi-fan motif includes four genes (A1, A2, B1 and B2), and is colored with dark green. FFL motif includes three genes (B3, C4 and D4), and is colored with pink. Figure S2. tSNE plots of human (A) and mouse (B) hematopoietic cells, colored by cell types (C) The co-expression subnetwork expressed in lymphoid progenitors (D) The co-expression subnetwork expressed in GMP progenitors Figure S3. (A) Curve of odds ratio for the enrichment of gene pairs in co-expression network in TF perturbation experiment versus all possible TF-TG pairs with different correlation cutoff. (B-G) Curves of odds ratio for the enrichment of gene pairs in co-expression network in annotation in STRING (B-C), FunCorp (D-E) and TTRUST (F-G) versus all possible TF-TG pairs with different correlation cutoff [file 12864_2020_7241_MOESM1_ESM.pdf]
