# Supplementary material for: Comprehensive network modeling from single cell RNA sequencing of human and mouse reveals well conserved transcription regulation of hematopoiesis
Source: BMC Genomics. 2020 Dec 29;21(Suppl 11):849. doi: 10.1186/s12864-020-07241-2 (PMC7771096; doi:10.1186/s12864-020-07241-2)
Supplement: Supplementary file 7 — Additional file 7: Supplemental file 3. 3-node and 4-node motifs identified by mfinder in GRN and cell type specific subGRNs [file 12864_2020_7241_MOESM7_ESM.pdf]

GRN

ID ZSCORE PVAL
38 41.08 0.000
0 1 1
0 0 1
0 0 0
----
204 25.41 0.000
0 0 1 1
0 0 1 1
0 0 0 0
0 0 0 0
----
206 101.60 0.000
0 1 1 1
0 0 1 1
0 0 0 0
0 0 0 0
----
472 11.83 0.000
0 0 0 1
1 0 1 1
1 0 0 0
0 0 0 0
----
906 11.67 0.000
0 1 0 1
0 0 0 1
1 1 0 0
0 0 0 0

2190 8.39 0.000
0 1 1 1
0 0 0 1
0 0 0 1
0 0 0 0

2252 51.77 0.000
----
0 0 1 1
0 0 1 1
0 0 0 1
0 0 0 0

2254 171.90 0.000
0 1 1 1
0 0 1 1
0 0 0 1
0 0 0 0

MEPsubGRN

ID ZSCORE PVAL
38 42.39 0.000
0 1 1
0 0 1
0 0 0
----
204 9.12 0.000
0 0 1 1
0 0 1 1
0 0 0 0
0 0 0 0
----
206 7.12 0.000
0 1 1 1
0 0 1 1
0 0 0 0
0 0 0 0
----
456 4.29 0.000
0 0 0 1
0 0 1 1
1 0 0 0
0 0 0 0
----
472 5.56 0.000
0 0 0 1
1 0 1 1
1 0 0 0
0 0 0 0
----
904 2.12 0.040
0 0 0 1
0 0 0 1
1 1 0 0
0 0 0 0
----
906 5.64 0.000
0 1 0 1
0 0 0 1
1 1 0 0
0 0 0 0

2252 4.92 0.000
0 0 1 1
0 0 1 1
0 0 0 1
0 0 0 0

LYMPHsubGRN

ID ZSCORE PVAL
38 57.60 0.000
0 1 1
0 0 1
0 0 0
----
206 96.94 0.000
0 1 1 1
0 0 1 1
0 0 0 0
0 0 0 0
----
472 10.43 0.000
0 0 0 1
1 0 1 1
1 0 0 0
0 0 0 0
----
906 24.88 0.000
0 1 0 1
0 0 0 1
1 1 0 0
0 0 0 0

2204 8.25 0.000
0 0 1 1
1 0 0 1
0 0 0 1
0 0 0 0

2252 65.66 0.000
0 0 1 1
0 0 1 1
0 0 0 1
0 0 0 0
----
204 3.83 0.000
0 0 1 1
0 0 1 1
0 0 0 0
0 0 0 0

2254 122.24 0.000

0 1 1 1
0 0 1 1
0 0 0 1
0 0 0 0

2506 16.76 0.000
0 1 0 1
0 0 1 1
1 0 0 1
0 0 0 0

HSCsubGRN

ID ZSCORE PVAL
38 27.75 0.000
0 0 1
0 0 0
----
78 2.94 0.000
0 1 1 1
0 0 1 0
0 0 0 0
0 0 0 0
----
92 6.21 0.000
0 0 1 1
1 0 1 0
0 0 0 0
0 0 0 0
----
204 26.19 0.000
0 0 1 1
0 0 1 1
0 0 0 0
0 0 0 0
----
206 11.47 0.000
0 1 1 1
0 0 1 1
0 0 0 0
0 0 0 0
----
408 6.35 0.000
0 0 0 1
1 0 0 1
1 0 0 0
0 0 0 0
----
472 3.16 0.020
0 0 0 1
1 0 1 1
1 0 0 0
0 0 0 0

2186 4.32 0.000
0 1 0 1
0 0 0 1
0 0 0 1
0 0 0 0

2252 8.11 0.000

0 0 1 1
0 0 1 1
0 0 0 1
0 0 0 0

GMPsubGRN

ID	ZSCORE	PVAL
38	9.56	0.000

0 1 1
0 0 1
0 0 0

204	5.81	0.000

0 0 1 1
0 0 1 1
0 0 0 0
0 0 0 0
